# microRNA‐221 rescues the loss of dopaminergic neurons in a mouse model of Parkinson's disease

**DOI:** 10.1002/brb3.2921

**Published:** 2023-02-16

**Authors:** Yufang Yao, Zhiyue Zhao, Fubo Zhang, Na Miao, Nan Wang, Xin Xu, Chaoping Yang

**Affiliations:** ^1^ Department 7 of Neurology Cangzhou Central Hospital Cangzhou Hebei China; ^2^ College of Mechanical and Electrical Engineering Cangzhou Normal University Cangzhou Hebei China; ^3^ Department 4 of Neurology Cangzhou Central Hospital Cangzhou Hebei China; ^4^ Department 1 of Traditional Chinese Medicine Cangzhou Central Hospital Cangzhou Hebei China

**Keywords:** Bax/caspase‐3 signaling, Bim, MiR‐221, Parkinson's disease

## Abstract

**Background:**

Parkinson's disease (PD) is one of the most common systemic neurodegenerative diseases and is related to the loss of dopaminergic neurons in the substantia nigra. Several studies verified that microRNA (miRNAs) targeting the Bim/Bax/caspase‐3 signaling axis is involved in the apoptosis of dopaminergic neurons in substantia nigra. In this study, we aimed to explore the role of miR‐221 in PD.

**Methods:**

To examine the function of miR‐221 in vivo, we used a well‐established 6‐OHDA‐induced PD mouse model. Then we conducted adenovirus‐mediated miR‐221 overexpression in the PD mice.

**Results:**

Our results showed that miR‐221 overexpression improved motor behavior of the PD mice. We demonstrated that overexpression of miR‐221 reduced the loss of dopaminergic neurons in the substantia nigra striatum by promoting their antioxidative and antiapoptosis capacities. Mechanistically, miR‐221 targets Bim, thus inhibiting Bim and Bax caspase‐3 mediated apoptosis signaling pathways.

**Conclusion:**

Our findings suggest miR‐221 participates in the pathological process of PD and might be a potential drug target and provide new insight into PD treatment.

## Graphical abstract

Predicted resting metabolic rate by equation has a reversely significant association with all‐cause mortality and poor functional outcome after ischemic stroke that indicates it seems to be a protective predictor of poor outcome of ischemic stroke as a metabolic proxy.

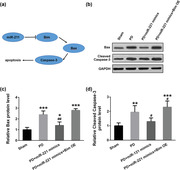

## 1 INTRODUCTION

Parkinson's disease (PD) is one of the most common systemic neurodegenerative diseases, resulting from structure and function loss of neuronal cells or even neuron death (Jiang et al., 2020; Mortal, 2021). PD influences ∼1% of the elder individuals over 65 years old (Massano & Bhatia, 2012). The clinical manifestations of PD include static tremors, muscle stiffness, impaired movement, postural instability, and bradycardia (Cao et al., 2017). Pathologically, the characteristics of PD are mainly related to Lewy body formation. The Lewy body consists of synaptic protein α‐synuclein and the dopaminergic neuron death in the substantia nigra (Hirsch et al., 1988). Several studies have reported that the Bax/caspase‐3 signaling pathway, which is involved in the translocation of Bax from the cytoplasm to the mitochondria, may contribute to the apoptosis of dopaminergic neurons in the substantia nigra (Perier et al., 2007; Zhang et al., 2019b). However, the intrinsic regulatory events of Bax/caspase‐3 signaling in PD are still incompletely known. Therefore, further studies are needed to unveil the molecular mechanism and characterize potential therapeutic approaches for PD.

Recent evidence has shown that microRNAs (miRNAs), a cluster of one strand, small and noncoding RNA, are associated with many neuronal disorders, including PD (Cao et al., 2017). Several miRNAs, like miR‐548d, miR224, and miR373, are identified as promising biomarkers for PD (Basak et al., 2016; Cardo et al., 2014). Among all these miRNAs, Ma et al. (2016) investigated that in PD patients, miR‐221 exhibited a negative correlation with the disease severity, indicating that miR‐221 can be used as a biomarker for detecting the developing stages of PD. However, the function of miR‐221 in PD remains unclear.

In this study, we found that miR‐221 was significantly downregulated in the plasma of PD patients. Then we demonstrated the role of miR‐221 in the 6‐OHDA induced PD mouse model. Our results showed that miR‐221 inhibited the activation of Bax/caspase‐3 signaling by restricting Bim, one of the newly identified proapoptotic members of the Bcl‐2 protein family (Cosialls et al., 2020), thereby reducing the apoptosis of dopaminergic neurons and contributing to the alleviation of PD. Our findings suggested that miR‐221 attenuated PD severity and might be a new therapeutic target for PD.

## 2 MATERIAL AND METHODS

### 2.1 Participants and blood samples

The study was under strict supervision, and the protocol was approved by the Ethical Committee of Cangzhou Central Hospital. A total of 70 PD patients and 72 healthy controls were recruited in this study. All the participants were required to sign informed written consent before the study started. The demographics and clinical characteristics, including sex, age, and family history, of the participants, were recorded the first time they came to the hospital.

### 2.2 Quantitative real‐time polymerase chain reaction (qRT‐PCR)

Total RNA of substantia nigra was extracted using Trizol reagent (Invitrogen, CA, USA). Then cDNA was synthesized immediately using reverse transcription kits (Tiangen, Beijing, China). GAPDH is used as the internal control. The supernatant of the participants’ plasma samples was used for miRNA isolation. qRT‐PCR was performed in a Biorad real‐time PCR instrument. The primer sequences are as follows: mmu‐miR‐221: F: CTG GTA GAG CTA CAT TGT CTG C; R: AAC TGG TGT CGT GGA GTC GG; Bim: F: CCC GGA GAT ACG GAT TGC AC; R: CAG CCT CGC GGT AAT CAT TTG. All data were analyzed in 2^−ΔΔCt^ method.

### 2.3 Mice and PD model establishment

Animal studies were approved by the Ethical Committee of Cangzhou Central Hospital. Twenty healthy C57BL/6J male mice (8 weeks, weighing 22–24 g) were housed under standard controlled conditions (∼23°C, 12:12 light‐dark cycle, 6:30 to 18:30) and were freely available to freshwater and chow.

For PD mouse model induction, 0.02% ascorbic acid sterile normal saline was utilized to prepare a dissolving solution containing 2 µL of 6‐hydroxydopamine (6‐OHDA; H4381, Sigma, MO, USA). Before induction, the mice were fixed on the stereotactic frame after anesthesia with 1.5% pentobarbital sodium. In order to block 6‐OHDA uptake in norepinephrine axons and terminals, desipramine and pargyline were administered to mice. Then the dissolved 6‐OHDA solution was injected into the substantia nigra of the mice with a flow rate of 0.5 µL/min. At the same time, the same volume of sterile saline containing 0.02% ascorbic acid was injected into the same area of the control group (Zhang et al., 2019a).

After that, the mice were randomly divided into 4 groups, including sham, PD, PD + miR‐221 mimics, and PD + miR‐221 mimics + BIM OE (overexpression). The sham and the PD groups were treated with lentivirus containing mir‐NC, the PD + miR‐221 mimics were treated with lentivirus containing miR‐221, and the PD + miR‐221 mimics + BIM OE were treated with lentivirus containing miR‐221 and BIM at the same time.

### 2.4 Mice behavior assessment

Rotation test: All mice were injected with apomorphine intraperitoneally at the dose of 0.5 mg/kg on the 14th day after establishing the PD model. Five minutes later, the contralateral hind limb of mice with substantia nigra injury was used as the support point, a standard rotation was defined as the head connected to the tail, and the body was bent into a circle. Each mouse's rotation behavior was observed and recorded, and the rotation times of mice within 30 min in each group were counted (Cai et al., 2020).

Stepping test: The forelimbs of the mice were placed on the table and applied with food pigment. Then raise the height of the table to let the mice support their body with their forelimbs. The experimenter grabs the tails of mice and slowly drags the mice backward to record the strides of both forelimbs. The mice were stroked and grasped for 3 min before the experiment to make it easier for them to adapt to the grasp (Barros et al., 2017). Contralateral step adjustment (%) = (contralateral steps/total steps) × 100%.

Cylinder test: The model mice were placed in a transparent cylinder, and the times of their forelimbs touching the wall within 5 min were recorded, including left, right, and bilateral forelimbs. The mouse was required to use the posterior limb as a supporting point, one side touched the wall first, and the other side touched the wall later was regarded as one complete case. The cylinder was wiped with ethanol after each mouse was tested. Contralateral utilization (%) = [(contralateral contacts + 1/2 contralateral contacts)/total contacts] × 100%. Before we push forward with the further experiments, mice without PD symptoms were excluded. For better results, the behavioral tests were carried out in the order of cylinder test, stepping test, and rotarod test. After each test, the animals rested for 3 h.

### 2.5 Immunohistochemistry

On the 14th day after 6‐OHDA administration, mice were anesthetized and fixed in the operation table, and their brains were perfused with 0.9% normal saline. The mouse brain were dissected immediately and fixed with 4% paraformaldehyde for 24 h, then were frozen in 30% sucrose solution (Jiang et al., 2020). Routine tissue sections and immunohistochemical experiments were carried out on the substantia nigra of mice. The primary antibody against TH (MAB318, Millipore, MA, USA) was diluted in the ratio of 1:2000 with TBST containing 5% BSA and incubated at 4°C overnight. The cross‐sectional area substantia nigra was measured with Image J (media control genetics, USA), and the number of TH + cells/square millimeter at higher magnification was counted. One slice out of every 6 adjacent slices, and six cross‐sections from rostral to caudal were selected from the whole substantia nigra of each mouse to calculate the TH+ neuron number.

### 2.6 Detection of the content of malondialdehyde (MDA), superoxide dismutase (SOD), and glutathione peroxidase (GSH‐Px)

Mice were sacrificed after the behavior test. The substantia nigra striatum was carefully separated from the mouse brain, weighed, and homogenized by ultrasound. The homogenized tissues were centrifuged at 4°C with the highest speed for 10 min, and the supernatant was collected. Coomassie brilliant blue staining was used to determine the concentration of protein. According to the manufacturers’ instructions, MDA, SOD, and GSH‐Px were measured with a spectrophotometer at 532, 550, and 422 nm, respectively (Nanjing Jiancheng Bioengineering Research Institute). The experiment was triply repeated.

### 2.7 Statistical analysis

The statistical analysis in the current study was performed utilizing Prism GraphPad. Data were expressed as mean (± SD) unless there was other statement. Data were analyzed by Student's *t*‐test, χ^2^ test, or one‐way ANOVA analysis followed by a post hoc test. *p* < .05 was considered statistically significant.

## 3 RESULTS

### 3.1 Patient baseline information

The baseline characteristics between the two groups showed no significant differences, regarding age (66.9 ± 8.1 years in the control group, 69.6 ± 10.2 years in PD patients), gender ratio (M38/F34 in the control group, M39/F31 in PD patients), smoking rate (16.7% in control group, 22.9% in PD patients), or drinking rate (29.2% in control group, 25.7% in PD patients) (Table 1). However, only 7 individuals in the control group have a family history, whereas 16 in PD patients (*p* value = .034). In addition, the UPDRS ADL in PD patients was 7.9 ± 4.3. 4 mL of fasting venous blood was collected from all the participants. The plasma samples were isolated by centrifugation for the following experiment.

**TABLE 1:** Demographics information and clinical characteristics of PD patients and healthy controls

	Controls (*n* = 72)	PD patients (*n* = 70)	*p* Value
Sex			
Female	34 (47.2)	31 (44.3)	.726
Male	38 (52.8)	39 (55.7)	
Age (years)	66.9 ± 8.1	69.6 ± 10.2	.084
UPDRS ADL	n/a	7.9 ± 4.3	/
Smoking			
Yes	12 (16.7)	16 (22.9)	.354
No	60 (83.3)	54 (77.1)	
Drinking			
Yes	21 (29.2)	18 (25.7)	.645
No	51 (70.8)	52 (74.3)	
Family history	7 (9.7)	16 (22.9)	.034

UPDRS, Unified Parkinson's Disease Rating Scale; ADL, activities of daily life.

### 3.2 The plasma level of miR‐221 was downregulated in PD patients

**FIGURE 1:**
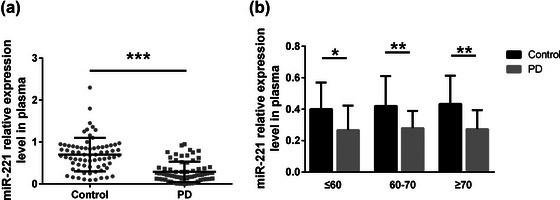
The relative expression level of miR‐221 in the plasma of individuals in the PD group and the control groups. (a) MiR‐221 expression was significantly decreased in PD patients (*n* = 70) in comparing of controls (*n* = 72). (b) Relative miR‐221 levels in PD patients are lower compared with healthy controls in different age group. *p < .05, **p < .01, ***p < .001.

The relative expression level of miR‐221 in the plasma of PD patients was markedly lower than that of the healthy control (*p* < .001) (Figure 1a). Concerning the relevance between miR‐221 expression level and participants’ age, PD patients at any age (≤60, 60–70, and ≥70) showed a decreased expression of miR‐221, compared with the healthy control. Notably, PD patients over 60 years old exhibited a much significantly lower plasma level of miR‐221 (*p* < .01) (Figure 1b).

### 3.3 MiR‐221 targets Bim in PD

Bim was reported to be a potential target of miR‐221 (Ye et al., 2016) and positively regulate Bax/caspase‐3 signaling pathway and promote apoptosis (Wang et al., 2016; Zhang et al., 2019b). On this basis, we further demonstrated whether miR‐211 affects PD through regulating Bim.

**FIGURE 2:**
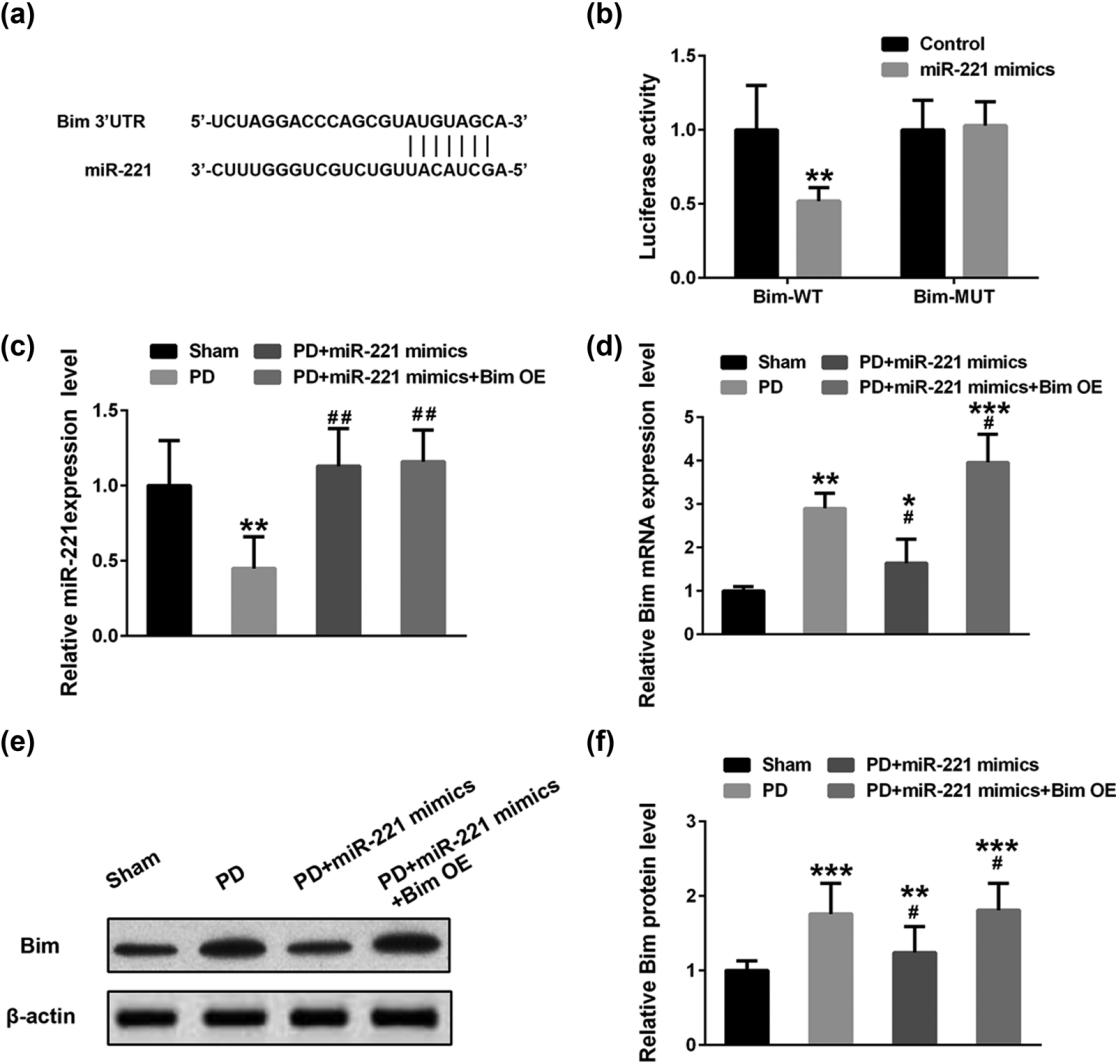
MiR‐121 targets to Bim. (a) The complementary binding site of miR‐221 in BIM 3′UTR. (b) A luciferase reporter assay was conducted to analyze the influence of miR‐211 on the transcription activity of Bim. Bim‐WT refers to wild type Bim 3′UTR and Bim‐MUT refers to the mutated miR‐211 binding sequence in the Bim 3′UTR. The luciferase activity of Bim‐WT treated with ctrl mimic miRNA was normalized as 1. The miR‐221 (c) and Bim mRNA (d) expression levels in the substantia nigra of mice were detected by qRT‐PCR. Bim protein expression was detected by Western blot (e) and relative expression level was analyzed. β‐actin was used for normalization. *n* = 3. *p < .05, **p < .01 compared with the sham group, #*p* < .05, ##*p* < .01 compared with the PD group.

To investigate the miR‐221 targeting site in Bim, we utilized the online bioinformatics website (http://www.targetscan.org). Figure 2a showed a miR‐221 seed sequence and its complementary binding site in the Bim 3′UTR region. Then we synthesized miR‐221 mimics, which are double‐stranded miR‐221‐like RNA fragments, and a control mimic, according to the sequence. Then we performed a luciferase reporter assay to analyze the influence of miR‐211 on the transcription activity of Bim. Our luciferase experiment revealed that the miR‐221 mimics could notably reduce the transcription activity of Bim (Figure 2b). However, miR‐211 mimics fielded to affect the luciferase activity when its binding sequence was mutated in Bim 3′ UTR. In the mouse PD model, we found that miR‐221 was significantly lower than that in the healthy control group; however, overexpression of miR‐221 mimics, as well as overexpression of miR‐221 mimics and Bim, can markedly rescue the expression of miR‐221 (Figure 2c, *F* = 68.8). Consistently, both the mRNA (Figure 2d, *F* = 34.2) and protein levels (Figure 2e and f, *F* = 149.6) of Bim elevated in the substantia nigra tissues of the PD mice. Overexpression of miR‐221 mimics blocked the expression of Bim. However, overexpression of Bim in vivo on this basis complements its expression level in the substantia nigra. These results suggested that Bim is a downstream target of miR‐221 in PD.

### 3.4 miR‐221 influences the behavior of PD mice by affecting Bim

**FIGURE 3:**
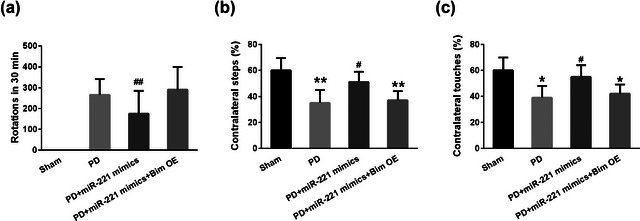
miR‐221 blocked the 6‐OHDA induced increase in rotations or the decrease in steps and touches of mice. (a) Number of rotations in each group after injection of apomorphine. Stepping test (b) and cylinder test (c) were used to evaluate motor defects of PD mice. *n* = 5. *p < .05, **p < .01 compared with sham group, #*p* < .05, ##*p* < .01 compared with PD group.

Fourteen days after 6‐OHDA induction, we detected the number of rotations, contralateral steps, and contralateral touches of mice in each group to evaluate the motor deficit of PD model mice. Compared to the healthy control group, the rotation number of PD mice increased markedly, and the contralateral steps and contralateral touches showed significant reductions. After miR‐221 mimics were overexpressed in PD mice, the number of rotations decreased, and the contralateral steps and contralateral touches elevated to a similar level to those in the healthy mice. Notably, when Bim was overexpressed in mice together with the miR‐221 mimics, Bim compromised the effects of miR‐221 on the number of rotations (Figure 3a), the contralateral steps (Figure 3b, *F* = 45.7), and contralateral touches (Figure 3c, *F* = 31.1) in PD mice. These results indicated that the function of miR‐221 in PD is attributed to targeting Bim.

### 3.5 miR‐221 inhibits dopaminergic neuron loss

**FIGURE 4:**
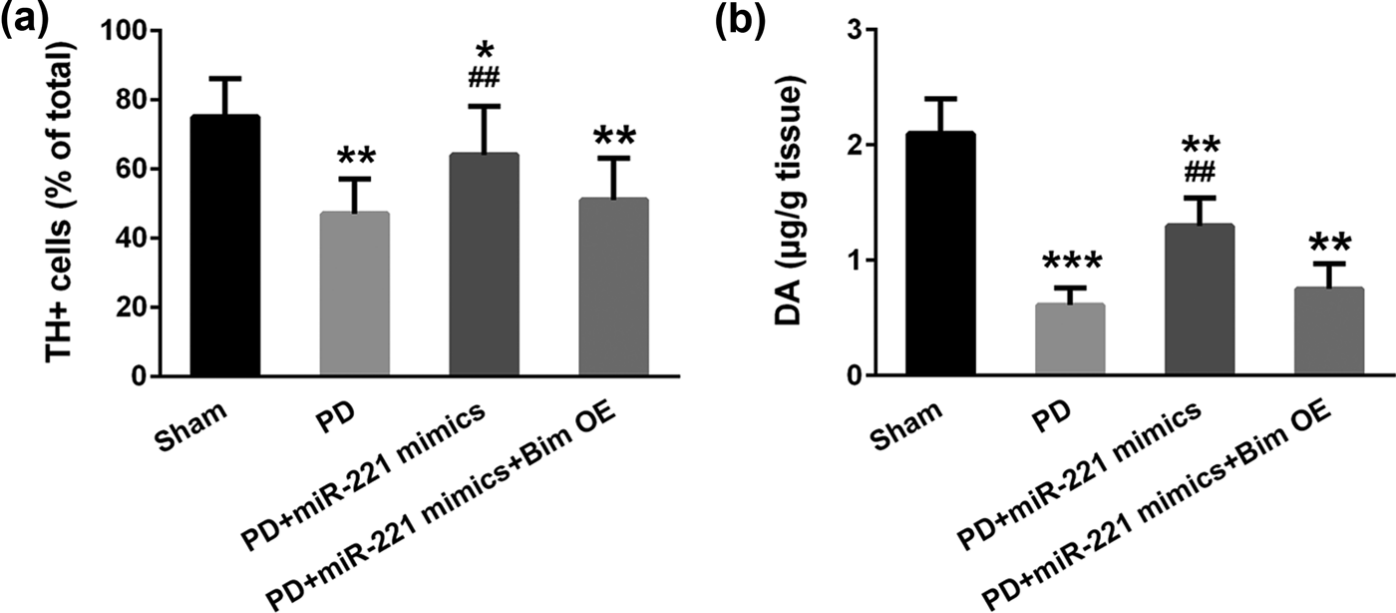
MiR‐221 protects the neurons from apoptosis. (a) Percentages of TH positive neurons in the substantia nigra region were quantified. Scale bar = 200 µm. (b) miR‐221 reverses striatal dopamine (DA) decreases induced by 6‐OHDA treatment. *n* = 5. *p < .05, **p < .01, ***p < .001 compared with the sham group, #*p* < .05, ##*p* < .01 compared with the PD group.

To determine the viability of the dopaminergic neurons, we conducted immunohistochemistry in the substantia nigra tissue sections to detect TH, a marker of viable dopaminergic neurons (Goncalves et al., 2020). As was shown in Figure 4a, in PD mouse substantia nigra, TH positive neurons were dramatically fewer than those in the sham group; lentivirus‐mediated overexpression of miR‐221 mimics in vivo significantly blocked the number of TH positive cells in the substantia nigra; and on this basis, overexpression of Bim blocked the increase of TH positive cells (*F* = 180.5). We also tested the striatal dopamine (DA) levels in the substantia nigra. Consistently, PD mice showed declined DA levels, and miR‐221 mimics rescued the 6‐OHDA induced DA decreases in PD mice. However, exogenously overexpression of Bim after miR‐221 mimics treatment abolished the effects of miR‐221 (Figure 4b). These data provided evidence that miR‐221 could prevent the injury of dopaminergic neurons.

### 3.6 MiR‐221 strengthens the antioxidant capacity in substantia nigra of mice

**FIGURE 5:**
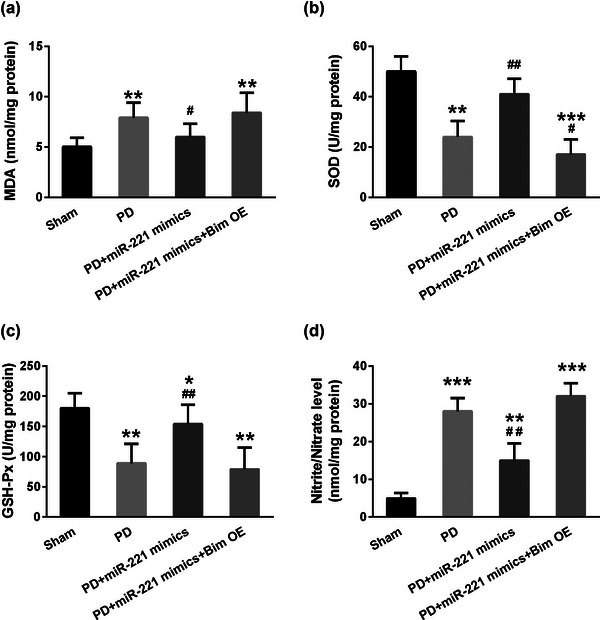
MiR‐221 alleviated the 6‐OHDA induced oxidative stress in mice. The plasma contents of MDA (a), SOD activity (b), GSH‐Px activity (c), and the total nitrite/nitrate level (d) in substantia nigra of mice in each group. *n* = 5. *p < .05, **p < .01, ***p < .001 compared with the sham group, #*p* < .05, ##*p* < .01 compared with the PD group.

To detect the antioxidant capacity of mice in each group, we measured the plasma contents of MDA, SOD, GSH‐Px, and Nitrite/Nitrate levels by Elisa assay. Compared with the sham group, the contents of MDA and the level of Nitrite/Nitrate were markedly increased in the PD group, while overexpressing miR‐221 mimics downregulated the two indexes. Notably, when Bim was overexpressed with miR‐221 mimics, the levels of MDA (Figure 5a, *F* = 86.2) and Nitrite/Nitrate (Figure 5d, *F* = 182.1) were significantly elevated. Regarding SOD and GSH‐Px activities, PD mice exhibited a dramatic decrease, whereas mice in the PD+miR‐221 mimics group showed a markedly lower level. However, mice in the PD+miR‐221 mimics + Bim OE group displayed significant increases in both SOD (Figure 5b, *F* = 110.5) and GSH‐Px (Figure 5c, *F* = 124.7) activities. These results indicated that miR‐221 modulates the antioxidative capacity of PD mice by targeting Bim.

### 3.7 miR‐221 downregulates Bax/caspase‐3 pathways via targeting Bim

**FIGURE 6:**
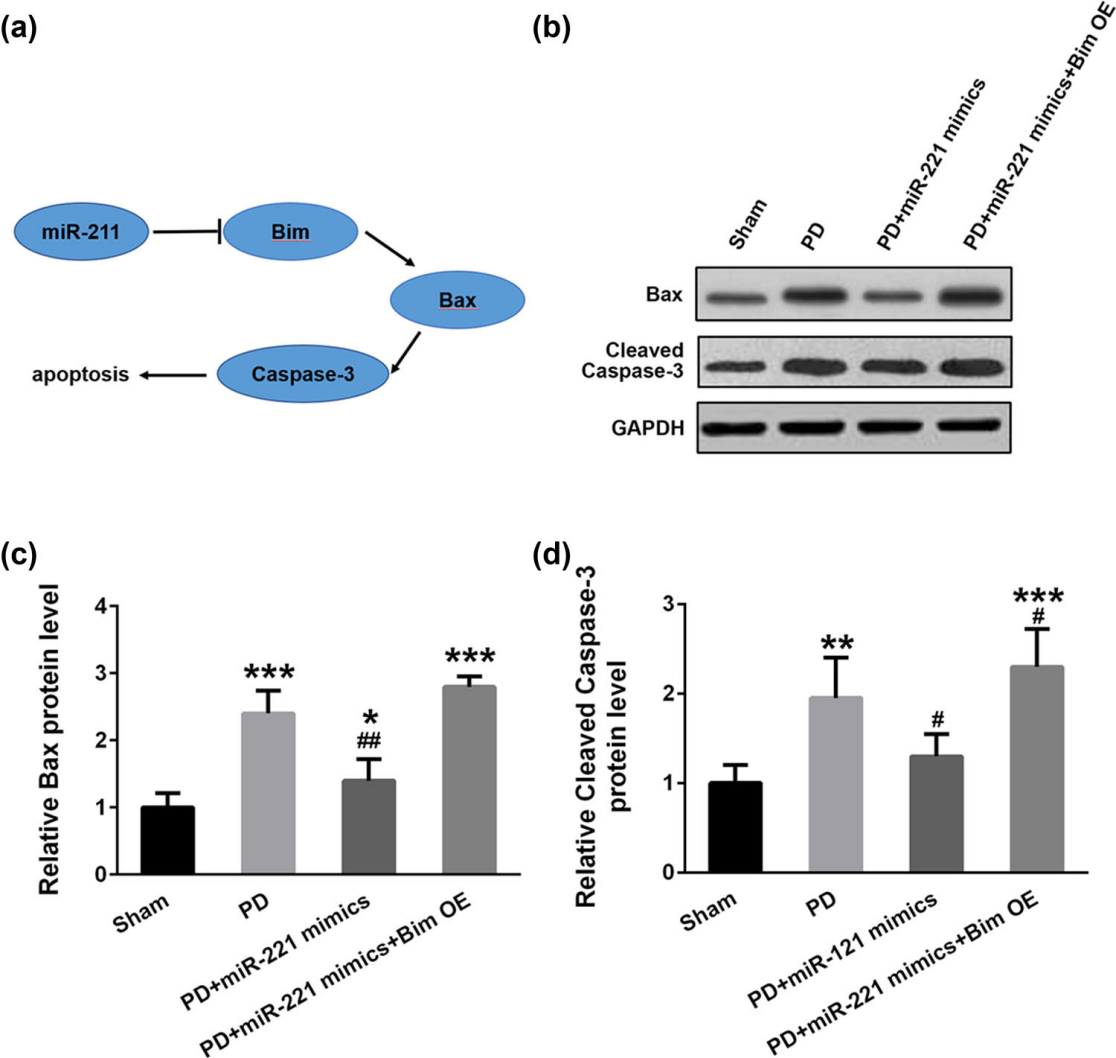
miR‐221 downregulates Bax/caspase‐3 pathways via targeting Bim. (a) Schematic representation of the major molecular mechanism that miR‐221 in cell apoptosis. (b) Western blot analysis of Bax/caspase‐3 pathways signaling pathway‐related proteins Bax and cleaved caspase‐3 in substantia nigra. Relative expression of Bax (c) and (d) cleaved caspase‐3 from Western blot were quantified. *n* = 5. **p < .01, ***p < .001 compared with sham group, #*p* < .05, ##*p* < .01 compared with the PD group.

Bim promotes apoptosis by regulating Bax/caspase 3 pathway (Kodama et al., 2011). Our previous results suggested that miR‐221 can target and regulate Bim; thus, we proposed a molecular mechanism diagram (Figure 6a). Then, we detected the effect of miR‐221 on the Bax/ caspase 3 apoptosis signaling pathway in mouse substantia nigra in each group. The Western blot assay verified that the expression of Bax in the PD group was significantly upregulated compared with the sham group. Overexpression of miR‐221 alleviated the upregulation of Bax expression, whereas overexpression of Bim rescued the downregulation of Bax caused by miR‐221 (Figure 6b and c, *F* = 73.3). Cleaved caspase‐3 showed the same trend as Bax in each group (Figure 6b and d, *F* = 107.5). These findings indicated that miR‐221 downregulated Bax/caspase‐3 pathways via targeting Bim.

## 4 DISCUSSION

The occurrence and development of Parkinson's disease mainly result from the apoptosis and loss of dopaminergic neurons in the substantia nigra compacta (Collaborators, 2018; Peng et al., 2018). The current PD treatment is mainly dopamine replacement therapy, which aimed to control motor symptoms (Cheong et al., 2019; Goncalves et al., 2020). However, the current treatment methods fail to interrupt or delay the progress of PD, nor improve the survival rate of dopaminergic neurons. In addition, this therapeutic approach may also lead to severe complications, such as cerebrovascular and cardiovascular diseases, and even death (Cuenca et al., 2019). Therefore, further studies should be conducted to deeper understand the molecular mechanisms of PD and eventually characterize potential biomarkers or curative approaches for PD. In this study, we found that the expression level of miR‐221 in the plasma of PD patients was significantly decreased. Coincidently, miR‐221 has been proved to be involved in the regulation of apoptosis signals (Ye et al., 2016). On this basis, we used the 6‐OHDA induced mouse PD model to explore the mechanism and feasibility of miR‐221 as a new target for PD diagnosis and therapeutic.

Previous studies have shown noteworthy differences in the abundance of miRNAs between PD patients and healthy controls, suggesting that miRNA can be used as a diagnostic marker of PD. Kim et al. (2007) reported that miR‐133b decreased in midbrain samples of patients with PD and played an important role in the differentiation and maintenance of dopamine neurons. Mir‐205 has also been confirmed to be significantly downregulated in PD, and the feasibility of mir‐205 as a diagnostic marker and treatment target is being further studied (Cho et al., 2013). Cai et al. reported that mir‐375 was also downregulated in PD. Overexpression of mir‐375 in PD mice can reduce dopaminergic neuron damage, oxidative stress, and inflammatory response through downregulating the expression of Sp1(Cai et al., 2020). Yang et al. (2019) determined that the plasma concentration of miR‐105‐5p in PD patients was dramatically higher than that in the healthy control group, and the expression level of miR‐105‐5p may be helpful to the identification of PD and other neurodegenerative diseases. In our current study, we demonstrated that overexpression of miR‐221 mimics in 6‐OHDA‐induced PD mouse significantly improve the motor ability of PD mice and increase the number of TH positive dopaminergic neurons, indicating that miR‐221 has the potential to inhibit neuronal damage and improve PD symptoms.

In our PD mouse model, we observed that miR‐221 was significantly lower in the substantia nigra tissues than that healthy mice. This result was consistent with the trend of motor ability and antioxidative abilities, such as content of MDA, SOD, and GSH‐Px, in each group of mice. Overexpressing miR‐221 mimics using lentivirus in the PD mice can markedly increase the expression of miR‐221, as well as mouse abilities of motor and antioxidation. Furthermore, we identified that the expression of Bim, which is a positive regulator of the Bax/Caspase‐3 signal, was modulated by miR‐221. In the substantia nigra tissue of the PD mice, Bim was notably upregulated. However, in the PD+miR‐221 mimics mice, both the mRNA and protein levels were reduced. Our data suggested that Bim is a downstream target of miR‐221 in PD. Our luciferase experiment confirmed that the miR‐221 mimics could effectively reduce the transcription activity of Bim. Since Bax/caspase‐3 is the direct target of Bim, we further analyzed the tissue expressions of Bax and caspase‐3 in each group of the mice. The result confirmed that the expression pattern of these two proteins is the same as Bim. These findings indicated that miR‐221 downregulates Bax/caspase‐3 pathways via targeting Bim.

Bim is a key molecule that regulates neuronal apoptosis. The nerve cells like sympathetic neurons while they were removed from nerve growth factor (NGF), cerebellar granule neurons (CGNs) lacking activity, and cortical neurons that were exposed to β‐amyloid peptide, are all regulated by Bim (Shi et al., 2005; Whitfield et al., 2001; Yao et al., 2007). Transcriptional regulation is one of the important mechanisms regulating the expression of Bim during neuronal apoptosis. Transcription factors, such as FOXO (Biswas et al., 2007), Egr‐1 (Xie et al., 2011), and JNK/c‐Jun pathway (Whitfield et al., 2001) have been reported to regulate BIM. Particularly, the mechanism by which Bim expression was regulated in the PD model has been unveiled to be related to the JNK/c‐jun singling activation and to the reduction of the translocation to mitochondrial field (Ponomarev et al., 2011). However, we observed that exogenous overexpression of miR‐221 can resist 6‐OHDA induced Bim upregulation in vivo, and there is growing evidence supporting the fact that Bim is regulated by microRNA in other diseases. For instance, mir‐363 promotes the survival of human glioblastoma stem cells by directly inhibiting BIM (Floyd et al., 2014), and mir‐24 inhibits Bim and the apoptosis process of mouse cardiomyocytes (Qian et al., 2011).

Inevitably, the current research has some limitations. The diagnostic criteria of PD and its closely related neurodegenerative diseases are extremely complex. Thus, further clinical studies are still needed to fully evaluate the feasibility of plasma miR‐221 levels in the precise diagnosis of differentiated PD. The evidence provided by our study emphasizes that miR‐221 regulates Bax/capase‐3 signaling pathway via acting on Bim, then participating in the pathological process of PD. Our findings suggested that miR‐221 might be a new target of PD therapy. However, further research is also needed to clarify the detailed mechanism of the miR‐221/BIM Bax/caspase‐3 axis.

## 5 CONCLUSION

In conclusion, our data showed that miR‐221 was dramatically downregulated in the substantia nigra tissues of the 6‐OHDA induced PD mouse model and the plasma of PD patients. Overexpression of exogenous miR‐221 blocked the loss of the dopaminergic neurons in PD mice. In addition, we demonstrated that miR‐221 regulates Bax/ccaspase‐3 signaling pathway by targeting Bim and participates in the regulation of neuronal apoptosis and survival during the pathogenesis of PD. Our work suggested that miR‐221 is a potential drug target for preventing dopaminergic neurons from apoptosis, which may provide a new reference for the diagnosis and clinical therapy of PD.

## CONFLICT OF INTEREST STATEMENT

The authors declared that they had no conflict of interest.

### PEER REVIEW

The peer review history for this article is available at https://publons.com/publon/10.1002/brb3.2921.

## Data Availability

Data will be made available upon reasonable request to the authors.
